# Secrets of safe laparoscopic surgery: Anaesthetic and surgical considerations

**DOI:** 10.4103/0972-9941.72593

**Published:** 2010

**Authors:** Arati Srivastava, Ashutosh Niranjan

**Affiliations:** Department of Anaesthesiology, Subharti Medical College, Meerut, India; Department of Surgery, Subharti Medical College, Meerut, India

**Keywords:** Obesity, pneumoperitoneum, pregnancy, previous surgery, safe laparoscopy

## Abstract

In recent years, laparoscopic surgery has gained popularity in clinical practice. The key element in laparoscopic surgery is creation of pneumoperitoneum and carbon dioxide is commonly used for insufflation. This pneumoperitoneum perils the normal cardiopulmonary system to a considerable extent. Every laparoscopic surgeon should understand the consequences of pneumoperitoneum; so that its untoward effects can be averted. Pneumoperitoneum increases pressure on diaphragm, leading to its cephalic displacement and thereby decreasing venous return, which can be aggravated by the position of patient during surgery. There is no absolute contraindication of laparoscopic surgery, though we can anticipate some problems in conditions like obesity, pregnancy and previous abdominal surgery. This review discusses some aspects of the pathophysiology of carbon dioxide induced pneumoperitoneum, its consequences as well as strategies to counteract them. Also, we propose certain guidelines for safe laparoscopic surgery.

## INTRODUCTION

Laparoscopic surgery is one of the most important diagnostic and therapeutic tools in the present surgical era. Since 1987, when the first laparoscopic cholecystectomy was successfully performed by Phillipe Mouret, this has become the gold standard. The benefits of minimal access techniques include less pain, early mobilization, minimal scar and shorter hospital stay, which have further increased its applications.[1] This minimally invasive procedure requires pneumoperitoneum for adequate visualization and operative manipulation. Systemic changes, in particular cardiopulmonary changes, also depend on the intra-abdominal pressure and the gas used. The major problems during laparoscopic surgery are related to the cardiopulmonary effect of pneumoperitoneum, systemic carbon dioxide absorption, venous gas embolism, unintentional injuries to intra-abdominal structures and patient positioning.[2,3] The goal of every laparoscopic surgeon should be to identify the risk factors, which may adversely affect anaesthetic as well as surgical outcome. We are briefly summarizing physiological changes associated with laparoscopic Surgery and listing some tips for laparoscopic surgeons.

## PHYSIOLOGICAL CHANGES DURING LAPAROSCOPIC SURGERY

Every laparoscopic surgeon must be aware of the physiological consequences of laparoscopic surgery. The proper understanding of changes in the body’s various systems during laparoscopic surgery will alleviate many complications. The physiological changes during laparoscopic surgery occur mainly due to two reasons: a) creation of pneumoperitoneum and b) position of patient during surgery.

The gas most commonly used for creation of pneumoperitoneum is carbon dioxide (CO_2_). The CO_2_-induced pneumoperitoneum exerts its physiological effects via two different mechanisms:

1. Mechanical effects relating to increased intraperitoneal pressure.
2. Chemical effect of CO_2_ used for insufflation.

The pneumoperitoneum leads to an increase in the intra-abdominal pressure with a consequent elevation of the diaphragm. This results in collapse of basal lung tissue ultimately causing decreased functional residual capacity (FRC), ventilation perfusion ratio (V/Q) mismatch, increase intrapulmonary shunting of blood which all lead to hypoxemia and increased alveolar arterial oxygen gradient [(A-a)DO_2_]. These consequences can be managed by increased frequency of mechanical ventilation with mild positive end-expiratory pressure (PEEP) and also by increasing fraction of inspired oxygen (FiO_2_) during laparoscopic surgery. Various studies support that a PEEP of 5 cm H_2_O should be considered essential during laparoscopic surgeries to decrease intraoperative atelectasis caused by pneumoperitoneum. This increases the FRC, thereby improving gas exchange and oxygenation.[4]

The cardiovascular changes occurring during laparoscopic procedure are because of both mechanical and chemical effects of CO_2_-induced pneumoperitoneum. The mechanical effect of pneumoperitoneum is compression of the inferior vena-cava, which causes reduction in venous return leading to decrease cardiac output and increase in the central venous pressure, resulting in increased vascular resistance in the arterial circulation.[5,8] These effects should be managed by infusing adequate fluid intraoperatively. Another effect is tachycardia, which is secondary to increased sympathetic discharge, hypercarbia and decreased venous return. The hypercarbia, acidosis, sympathetic stimulation from decreased venous return and vagal stimulation by stretching of peritoneum also disturb the cardiac rhythm. Moderate to severe hypercarbia can results in premature ventricular contractions, ventricular tachycardia and even ventricular fibrillation. Vagal stimulation may also cause bradyarrythmias. These effects can be prevented by minimizing the intra-abdominal pressure (not above 12 mm of Hg) and proper preoperative hydration and monitoring the end-tidal CO_2_ (et-CO_2_). Increased intra-abdominal pressure also reduces the visceral blood flow. The clinical significance or diminished blood flow is not clear, but it can be prevented by preoperative hydration.

During the laparoscopic procedure the position of the patient is either in Trendelenburg or in Reverse Trendelenburg. These positions have an impact on the cardiopulmonary function. In Trendelenburg position, there is an increase preload due to an increased in the venous return from lower extremities. This position results in cephalic shifting of viscera, which accentuates the pressure on the diaphragm. In case of reverse Trendelenburg position, pulmonary function tends to improve as there is caudal shifting of viscera, which improves tidal volume by decrease in the pressure on the diaphragm. This position also decreases the preload on heart and causes a decreased in the venous return leading to hypotension. The pooling of blood in the lower extremities increases the stasis and predisposes the deep vein thrombosis (DVT).

## ANAESTHETIC MANAGEMENT

The aim of anaesthetic management of patients undergoing laparoscopic surgery should be to allow the physiological changes during surgery with minimal effects on body’s vital systems and rapid recovery from anaesthesia with minimal residual effects. All these changes can be detected early by monitoring the electrocardiogram, noninvasive arterial pressure (NIBP), airway pressure, pulse oximeter (SpO_2_), et-CO_2_ concentration, peripheral nerve stimulation and body temperature. The urine output should also be monitored in patients with compromised cardiopulmonary function. The urinary catheterization also minimizes the risk of bladder injury during port insertion.

Atropine should be used judiciously, as it prevents vagal-stimulated bradyarrythmia but it also increases the risk of tachyarrhythmia.[9] Anxiolytics, such as the benzodiazepines may be prescribed in anxious patients the night before the surgery. Since postoperative nausea and vomiting (PONV) is a predicted complication of laparoscopic surgery, use of ondensetron is recommended preoperatively. The choice of anaesthetic technique for abdominal laparoscopic surgery is general anaesthesia with muscle paralysis, tracheal intubation and intermittent positive pressure ventilation (IPPV) with moderate tidal volume and addition of a PEEP of 5 cms of water.[10] A newer option to the anaesthetic technique is the use of a laryngeal mask airway (LMA) keeping the patient in spontaneous respiration. The stomach should be deflated by Ryle’s tube aspiration to avoid the risk of gastric injury during trocar insertion. The nitrous oxide has the ability to produce bowel distension, so its use during laparoscopy is controversial. Halothane increases the incidence of arrhythmia during laparoscopic surgery, especially in the presence of hypercarbia.[11] Isoflurane is the preferred volatile anaesthetic agent as it has less arrhythmogenic and myocardial depressant effects. Epidural anaesthesia has been used for outpatient gynaecological laparoscopic procedures to reduce complications and shorten recovery time after anaesthesia.[12] Some studies reveal that ventilation with a large tidal volume of 12-15 ml/kg prevents progressive alveolar atelectasis and hypoxaemia and allows for more effective alveolar ventilation and carbon dioxide elimination,[13] but others are in support of ventilation with moderate tidal volume and addition of PEEP. The adequacy of ventilation can be evaluated by measuring the gradient between PaCO_2_ and P_E_CO_2_ (tension of CO_2_ in expired air). In healthy patients under general anaesthesia, it is between 2 mmHg and 9 mmHg;[14] however, for patients with compromised cardiopulmonary function, the gradient between PaCO_2_ and P_E_CO_2_ may become high and unpredictable. So, direct estimation of PaCO_2_ by arterial blood gas analysis may be necessary to detect hypercarbia.[15] A P_E_CO_2_ monitor is also valuable for early detection of venous gas embolism.[16] An airway pressure monitoring should be done regularly in anaesthetized patients receiving IPPV, as high airway pressure alarm can aid in detection of excessive elevation of intra-abdominal pressure.[17]

Every patients undergoing laparoscopic surgery should be evaluated preoperatively for any:

1. Risk of anaesthesia,
2. Limiting factors for pneumoperitoneum, and
3. Coagulopathy disorders.

A detailed cardio-pulmonary history should be obtained to avoid complications during or after surgery.

- History of pulmonary disease: Whether the patient is suffering from any diseases which can decrease the pulmonary compliance. Even obstructive pulmonary diseases can interfere with gaseous exchange, resulting in an accentuation of hypercarbia state.
- History of cardiac diseases: As hypercarbia and pneumoperitoneum-induced peritoneal stretching stimulates sympathetic nervous system, even mild chronic hypertension can precipitate relative hypovolemia and hypotension. Thus proper history of hypertension and cardiac illness should be evaluated thoroughly.

Patients with previous abdominal surgery, obesity and pregnancy should be planned for laparoscopic surgery after careful preoperative evaluation. All the above conditions are not absolute contraindication for surgery. In case of patient with previous abdominal surgery, the only contraindication is a documented evidence of frozen abdomen.[18] Although these patients do not need any extra care from anaesthetic point of view, there is an increased chance of conversion to an open laparotomy and additional ports may be required for adhesiolysis. Initial port placement should be well away from all abdominal scars. The right or left upper quadrant in the midclavicular line is the safe starting point. Port entry can be made either by Veress needle entry with blind trocar insertion or open/Hasson entry with blunt-tip trocar or by optical trocar, if available.

Pregnancyis also not an absolute contraindication for laparoscopic surgery.[19] The laparoscopic surgery, if necessary, should be performed after first trimester and patient should be under proper care of obstetrician and uterine relaxant drugs. The port should be placed at a site such that it avoids injury to the gravid uterus. The initial port placement should be made by open/Hasson technique to avoid the risk of uterine injury. There is increase risk of development of fetal acidosis. This problem should be managed by maintaining et-CO_2_ between 25 and 33 by changing minute ventilation. The arterial blood gas monitoring should be considered as a special tool in these patients.

Obesity, when BMI is over 30, may be associated with co-morbidities such as cardio-pulmonary or metabolic disorders.[20] In these patients, an open Hasson technique is preferred to Veress needle entry. Extra long instruments may be needed in these patients, as they have very thick subcutaneous layer of fat. Proper visualization of intra-abdominal contents needs proper elevation of the anterior abdominal wall. This may result in increased pneumoperitoneum pressure up to 15–20 mmHg, so complete muscle relaxation should be provided during surgery. This high intra-abdominal pressure can result in hypercarbia, so these patients should be under strict monitoring of et-CO_2_ Any rise in et-CO_2_ should be managed by desufflation of abdomen and by putting the patient in reverse Trendelenburg position. Obesity is an independent risk factor for perioperative DVT formation, and therefore it is prudent to use compression pneumatic device along with subcutaneous heparin in these patients, unless contraindicated.

## CONCLUSION

Laparoscopic surgery, a modern surgical technique, has gained popularity over conventional abdominal surgery. There are a number of advantages of laparoscopic surgery as compared to an open procedure. These include reduced pain due to smaller incisions and minimal blood loss and shorter recovery time. The key element in laparoscopic surgery is the creation of pneumoperitoneum, which is generally made by CO_2_. The major problems during laparoscopic surgery are related to CO_2_-induced pneumoperitoneum, which can affect the cardiopulmonary function, systemic carbon dioxide absorption, extraperitoneal gas insufflation, venous gas embolism, unintentional injuries to intra-abdominal structures and patient positioning. Additional problems may occur in the obese, the pregnant ladies and in those who have had previous surgery. These problems can be averted if certain precautions have been kept in mind. These are:

- All the cardiopulmonary-compromised patients should be assessed preoperatively by a physician or a cardiologist. They are not contraindications for laparoscopic surgery. High-risk consent with intensive monitoring is mandatory to prevent mishaps.
- Informed consent for risk of anaesthesia in cardiopulmonary-compromised patients, additional port placement in case of previously operated patient, risk of abortion or preterm delivery in case of pregnant women should be explained.
- Lower pressure pneumoperitoneum (10–12 mmHg) with proper hydration of patient can prevent the consequences of preload and afterload on cardiac function.
- Minimize the operating time by taking the help of experienced person.
- Using helium or nitrous oxide gas for the creation of pneumoperitoneum, if available in cardiopulmonary-compromised patients.
- Measuring the et-CO_2_ and arterial blood gas analysis, especially in cardiopulmonary-compromised patients and pregnant women to avoid fetal acidosis.
- Extra long trocar and instruments may be needed in obese patients. Precaution to prevent DVT should be taken in these patients.
- First port placement for creation of pneumoperitoneum in previously operated patients or in pregnant women should be done by either open/Hasson technique or by optical technique. This port should be away from previous scar and gravid uterus.
